# Cathelicidins and the Onset of Labour

**DOI:** 10.1038/s41598-019-43766-7

**Published:** 2019-05-14

**Authors:** Sara R. van Boeckel, Lenka Hrabalkova, Tina L. Baker, Heather MacPherson, Lorraine Frew, Ashley K. Boyle, Brian J. McHugh, Kirsten Wilson, Jane E. Norman, Julia R. Dorin, Donald J. Davidson, Sarah J. Stock

**Affiliations:** 1 Tommy’s Centre for Maternal and Fetal Health at the MRC Centre for Reproductive Health, University of Edinburgh, QMRI, Edinburgh, United Kingdom; 2 University of Edinburgh Centre for Inflammation Research, QMRI, Edinburgh, United Kingdom

**Keywords:** Acute inflammation, Reproductive biology, Translational research

## Abstract

Preterm birth, defined as delivery before 37 weeks of gestation, is the leading cause of neonatal mortality and morbidity. Infection and inflammation are frequent antecedents of spontaneous preterm birth. Cathelicidin, an antimicrobial host defence peptide, is induced by infection and inflammation and although expressed in the reproductive tract and fetal tissues, its role in the pathogenesis of spontaneous preterm birth is unknown. Here we demonstrate that cathelicidin expression is increased at RNA and protein level in the mouse uterus in a model of inflammation-induced labour, where ultrasound guided intrauterine injection of lipopolysaccharide (LPS) at E17 stimulates preterm delivery within 24 hours. Cathelicidin-deficient (*Camp*^−/−^*)* mice are less susceptible to preterm delivery than wild type mice following intrauterine injection of 1 μg of LPS, and this is accompanied by a decrease in circulating IL-6, an inflammatory mediator implicated in the onset of labour. We also show that the proportion of cathelicidin expressing cells in the myometrium is higher in samples obtained from women in labour at term than pre-labour. Together, these data suggest that cathelicidin has roles in mediating pro-inflammatory responses in a murine model of inflammation-induced labour, and in human term labour.

## Introduction

Mechanisms controlling the onset of human labour are not well understood and disorders in the timing of labour onset are a major public healthcare burden. Spontaneous preterm labour is the most common cause of preterm birth (PTB) (delivery before 37 completed weeks of gestation). PTB is the leading cause of neonatal or infant death worldwide, and results in morbidities that can persist into adulthood^1^. The incidence of PTB ranges from 5% to 18% depending on the healthcare setting, with the incidence rising in many countries^2^. While approximately 30% of PTBs are indicated by maternal and/or fetal conditions, 70% of PTBs are spontaneous^2^.

Cathelicidins are a family of antimicrobial host defence peptides, a highly conserved component of the innate immune system. They are predominantly produced by epithelial cells, neutrophils and other inflammatory cells and are best known for their ability to act as broad-spectrum antimicrobial agents^3,4^. Cathelicidins also have functions in immunomodulation^5^, chemotaxis^6^, wound healing^7^ and angiogenesis^8^. This wide range of functions are believed to be mediated by an ability to activate multiple receptors, triggering different downstream pathways in a spatio-temporal manner that is dependent on the cell type and physiological context^3^. Humans and mice each express a single cathelicidin gene. In humans, *CAMP* encodes a precursor peptide (hCAP-18) that is either immediately released or stored intracellularly in neutrophil secondary granules. The precursor peptide is cleaved at the C-terminal by proteases to produce an active peptide called LL-37^3,9^. LL-37 is abundant in the female reproductive tract^10^, fetal skin, vernix caseosa and in the amniotic fluid^11^. The murine orthologue, cathelicidin-related antimicrobial protein (mCRAMP) is encoded by the gene *Camp*. It is produced by the same cell types as in humans and is believed to have similar functions to that of LL-37^3,12^.

Interestingly, the role of cathelicidin in pregnancy is unknown. We have recently shown that it can potentiate protective inflammation in response to infection *in vivo*^13^ suggesting that expression of this peptide could modulate inflammatory processes in the context of spontaneous PTB, which is often preceded by infection^2,14^.

Inflammation is recognized as a key feature of both normal term labour and PTB in humans^2,14–19^, characterised by infiltration of immune cells into gestational tissues and the release of inflammatory mediators that promote cervical ripening and myometrial activation^20,21^. Circulating progesterone levels remain high throughout parturition, and activation of inflammation in the uterus is thought to have a dominant role in promoting the change from uterine quiescence to a contractile phenotype. In most other mammals including mice, progesterone levels fall immediately prior to parturition. However, an intrauterine injection of lipopolysaccharide (LPS) is sufficient to activate inflammation and overcome the repressive effects of high circulating progesterone concentrations, inducing PTB^19,22^. This has become an established model for parturition research^22,23^.

In this study we used *Camp*^−/−^ mice, which lack the murine cathelicidin peptide, mCRAMP, to determine whether cathelicidin plays a role in the onset of PTB using an established LPS-induced PTB mouse model^22^. To determine if human cathelicidin levels change in response to labour we investigated mRNA levels of *CAMP* in myometrial samples from women obtained at caesarean section either before labour onset, or during labour; at preterm or at term gestations.

## Results

### Intrauterine LPS injection induces mCRAMP expression in the mouse uterus at mRNA and protein level

We have previously shown that intrauterine administration of 20 μg LPS at gestation day 17 induced PTB in wild type C57Bl/6 mice, with an increase in pro-inflammatory cytokines and chemokines at the maternal-fetal interface^22^. To determine the minimal dosage required to induce PTB, an LPS dose response was performed in wild type mice ranging from 0.3–20 μg/dam (Supplementary Fig. S1). 1 μg LPS was the lowest dose found to induce PTB showing a similar time to delivery as 20 μg LPS. However, 1 μg LPS showed a significantly reduced expression of inflammatory genes *Cxcl1* and *IL-6* compared to 20 μg LPS (Supplementary Fig. S2).

**Figure 1:**
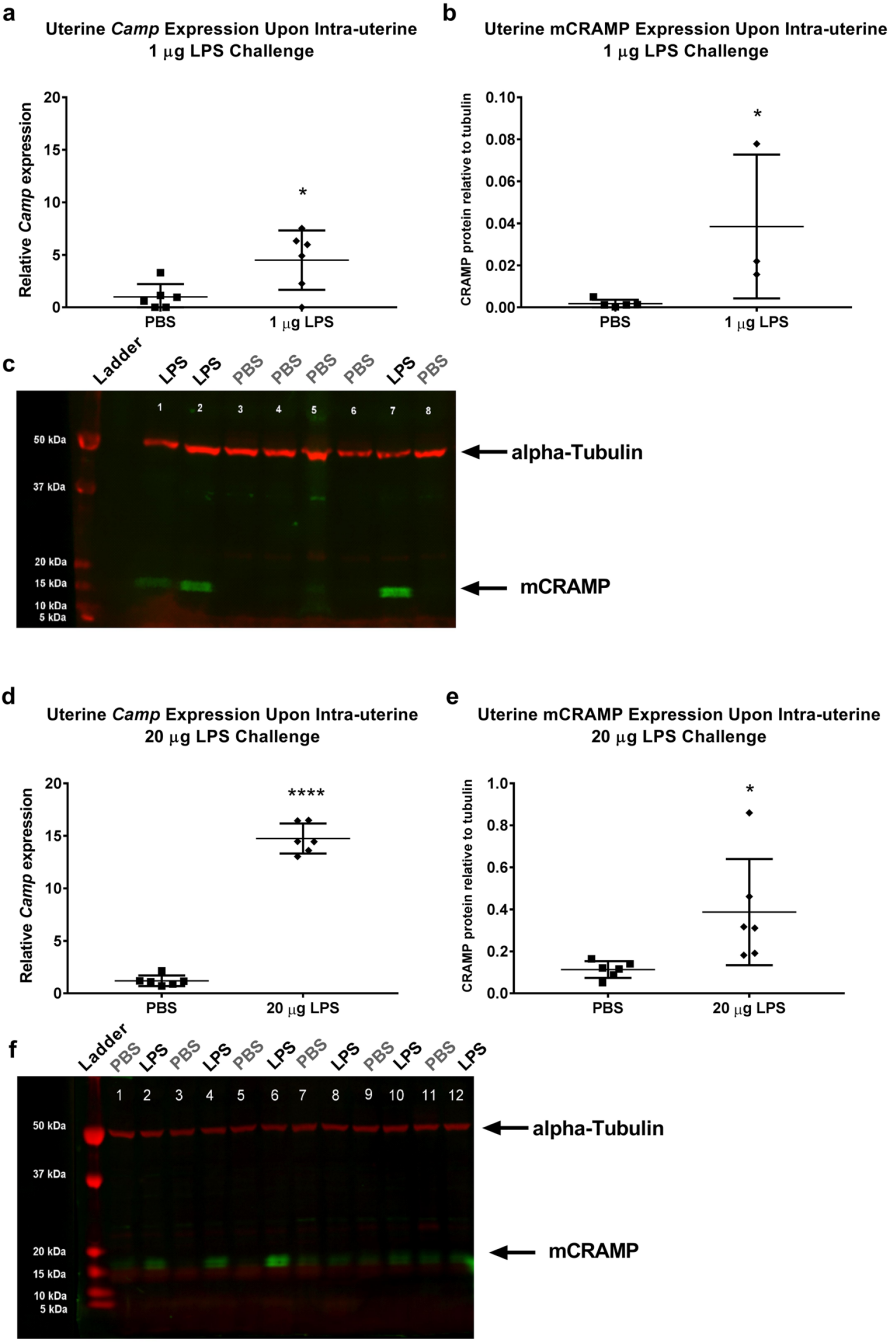
*Camp* and mCRAMP expression is significantly increased in the mouse uterus after 1 µg and 20 µg intrauterine LPS injection. Real-time PCR analysis of relative *Camp* mRNA expression after 1 µg (**a**) or 20 µg (**d**) intrauterine LPS or PBS injection. Relative mCRAMP protein levels after 1 µg (**b**) or 20 µg (**e**) intrauterine LPS or PBS injection as quantified by Western Blot analysis (**c**, **f**). mCRAMP protein levels were normalised against housekeeping alpha-Tubulin (50 kDa). The mCRAMP protein band is indicated by a black arrow at approximately 18 kDa. Unpaired t-test (*p < 0.05, ****p = 0.0001). Data presented as mean ± SD. The full-length blots with mCRAMP expression are presented in Supplementary Figs S3 and S4.

**Figure 2:**
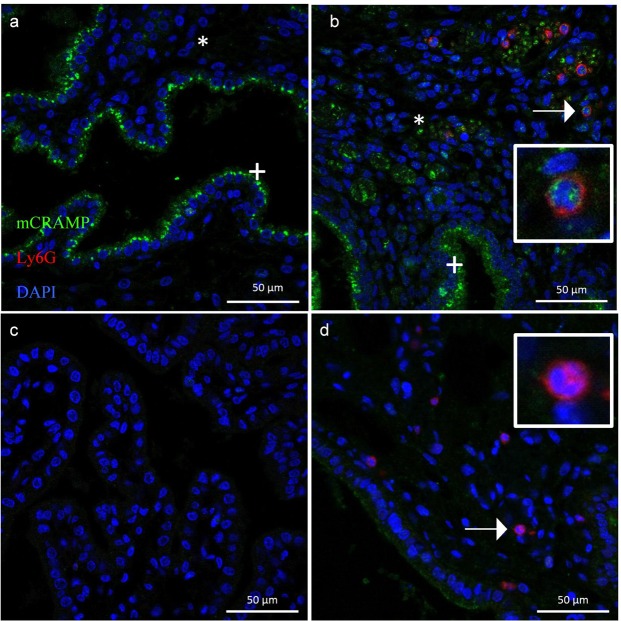
Representative images of mCRAMP expression in the mouse uterus. (**a**) mCRAMP (green) is present in uterine epithelium (+) but absent in the stromal compartment (*) following a control PBS injection. No Ly6G positive cells (red) (neutrophils) can be found. (**b**) Following a 1 µg LPS injection, upregulated mCRAMP is present in the epithelium (+) and stromal compartment (*), with expression shown in neutrophils (red) as indicated by arrow and magnified in the inset panel. (**c**) Secondary antibody-only negative control. (**d**) *Camp*^−/−^ uterus showing an absence of mCRAMP (green). mCRAMP negative neutrophil (red) is indicated by arrow and magnified in the inset panel.

We found that *Camp* mRNA expression and mCRAMP peptide levels were increased in the mouse uterus 6 hours after an intrauterine injection of both a 1 μg and 20 μg dose of LPS (Fig. 1). Immunofluorescence showed that mCRAMP is found in the uterine epithelium in PBS-treated control mice (Fig. 2a). Following a 1 μg LPS injection, mCRAMP was found in the uterine epithelium, stromal compartment and neutrophils as confirmed with dual staining with Ly6G (Fig. 2b).

### *Camp*^*−/−*^ mice are less susceptible to LPS – induced PTB

**Figure 3:**
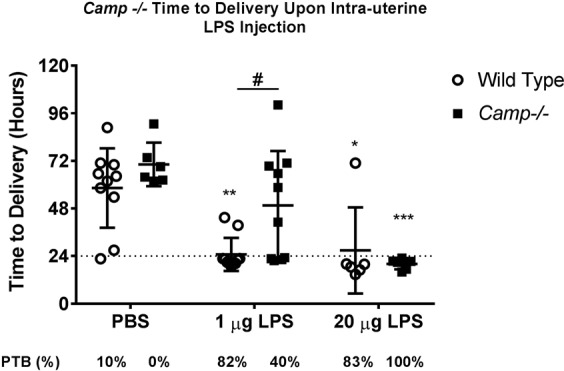
Time to delivery in *Camp*−/− and wild type mice after intra-uterine PBS or LPS injection. Time to delivery of first pup after intra-uterine injection with PBS, 1 µg or 20 µg of LPS in cathelicidin-deficient mice (*Camp*^−/−^*)* and wild type (C57BL/6J) mice. Significance representing LPS treated mice compared to PBS controls from same genotype (*p < 0.05, **p < 0.01, ***p = 0.001), ^#^p < 0.05 difference between genotype in the same treatment group, Two-way ANOVA). No symbol signifies no significant difference between the groups (p > 0.05). Preterm Birth (PTB) rates, defined as delivery before 24 hours rates in percentage are shown below. Significance is calculated by using Fisher’s Exact test.

To assess whether LPS-induced cathelicidin could mediate PTB, *Camp*^−/−^ and wild type mice were assessed after an intrauterine injection of either 1 μg or 20 μg of LPS, or PBS (negative control) (Fig. 3). Intrauterine injection of 1 μg LPS reduced time to delivery compared to PBS in wild type (25 ± 8.4 hrs following 1 μg LPS vs. 58 ± 20 hrs following PBS; p = 0.002) but not *Camp*^−/−^ mice (50 ± 27.4 hrs following 1 μg LPS vs. 70 ± 11 hrs following PBS; p = 0.253), with less PTB (defined as delivery within 24 hours of intrauterine injection) in *Camp*^−/−^ mice than wild type mice (82% vs. 40%; p = 0.015). In contrast, 20 μg LPS reduced the time to delivery similarly in wild type and *Camp*^−/−^ mice, with no difference in PTB rates (83% and 100%, respectively, p > 0.999).

### *Camp*^*−/−*^ mice have significantly decreased circulating IL-6 following intrauterine LPS injection

**Figure 4:**
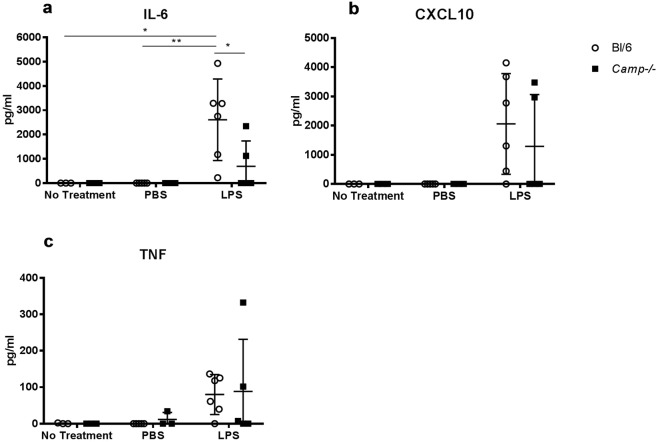
IL-6, CXCL10 and TNF production in the maternal circulation after LPS injection. 6 hours after intrauterine injection with 1 µg LPS, PBS or no treatment at the same gestation. (**a**) Circulating interleukin-6 (IL-6) was significantly greater in the wild type mice (C57Bl/6J) (N = 6) compared to *Camp*^−/−^ mice (N = 5, *p = 0.045) upon LPS treatment. (**b**, **c**) No significant differences were seen in CXCL10 or TNF production. Data presented as mean ± SD, Two-way ANOVA, Tukey’s multiple comparison test.

Having shown that *Camp*^−/−^ mice are less susceptible to LPS-induced PTB, we explored differences in maternal cytokine levels and uterine inflammatory gene expression following 1 μg LPS injection in wild type and *Camp*^−/−^ mice. Maternal blood of *Camp*^−/−^ mice had significantly lower circulating IL-6 compared to wild type following LPS injection (694 ± 1045 pg/ml vs. 2608 ± 1678 pg/ml; p = 0.045) (Fig. 4). No differences were seen in circulating CXCL10 or TNF levels (p = 0.996 and p > 0.999, respectively).

**Figure 5:**
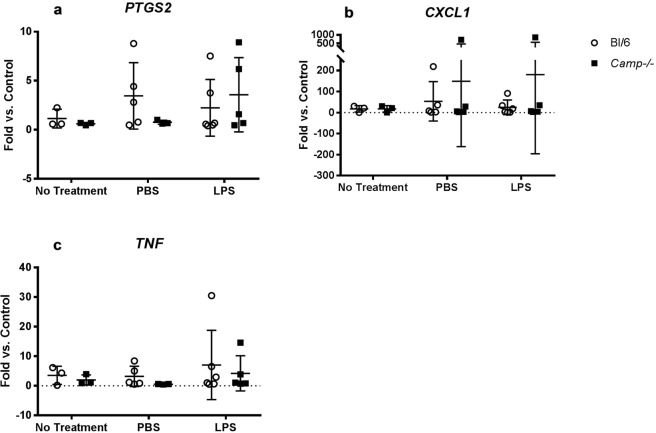
*PTGS2*, *CXCL1* and *TNF* gene expression in the mouse uterus after LPS injection. 6 hours after intra-uterine injection with 1 µg LPS, PBS or no treatment, at the same gestation. No significant differences were seen in *PTGS2* (**a**) *CXCL1* (**b**) or *TNF* (**c**) expression between wild type and *Camp*^−/−^ mice. Data presented as mean ± SD, Two-way ANOVA, Tukey’s multiple comparison test.

The gene expression of prostaglandin-endoperoxide synthase 2 (*PTGS2*), chemokine (C-X-C motif) ligand 1 (*CXCL1*) and tumor necrosis factor (*TNF)* were unaltered in the uteri of *Camp*^−/−^ mice compared to wild type mice, irrespective of whether 1 μg of LPS, PBS or no treatment was given (Fig. 5).

### *CAMP* gene expression and quantification of LL-37 positive cells in human myometrial samples

Myometrial samples were classified into term pre-labour caesarean section (Term No Labour [TNL]), term labour (TL), preterm pre-labour caesarean section (Preterm No Labour [PTNL]) preterm labour (PTL) (Table 1). Labour was defined as presence of regular uterine contractions leading to progressive cervical dilatation.

**Table 1:** Clinical demographics of human myometrial sample cohort.

	Term No Labour	Term Labour	Preterm No Labour	Preterm Labour
Number of myometrial samples collected	14	13	13	5
Maternal age at collection	37 (24–44)	32 (22–39)	35 (24–41)	31 (20–36)
Indication for CS	Elective CS (included previous CS, breech, placenta previa, previous traumatic delivery)	Emergency CS (included failure to progress and/or suspected fetal compromise, planned CS but presented in labour)	Emergency CS (included suspected fetal compromise, pre-eclampsia, maternal medical condition)	Emergency CS (included suspected fetal compromise in labour, antepartum haemorrhage in labour, breech presentation in labour)
Gestational age at time of CS (weeks.days)	39.1 (39–40.1)	40.4 (39–41.3)	33.9 (27.4–36.4)	35.1 (33.4–36.4)
Cervical dilation at time of CS (cm)	—	5 (2–10)	—	5 (1–5)

Data is presented as median (range). CS: Caesarean section.

**Figure 6:**
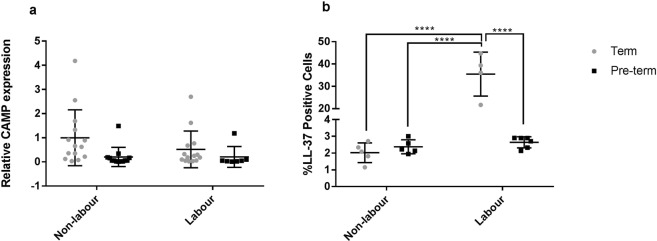
Relative *CAMP* expression and percentage of LL-37 positive cells in TNL, PTNL, TL, and PTL myometrium. (**a**) No difference in relative *CAMP* expression relative to *β-Actin* was found between TNL, PTNL, TL, and PTL myometrium. (**b**) The percentage of LL-37 positive cells was found to be significantly higher in TL myometrium in comparison to TNL, PTNL and PTL myometrium. Regular 2-way ANOVA with a Tukey’s multiple comparisons test was used to determine statistical significance. ****= p < 0.001. Data presented as mean ± SD.

**Figure 7:**
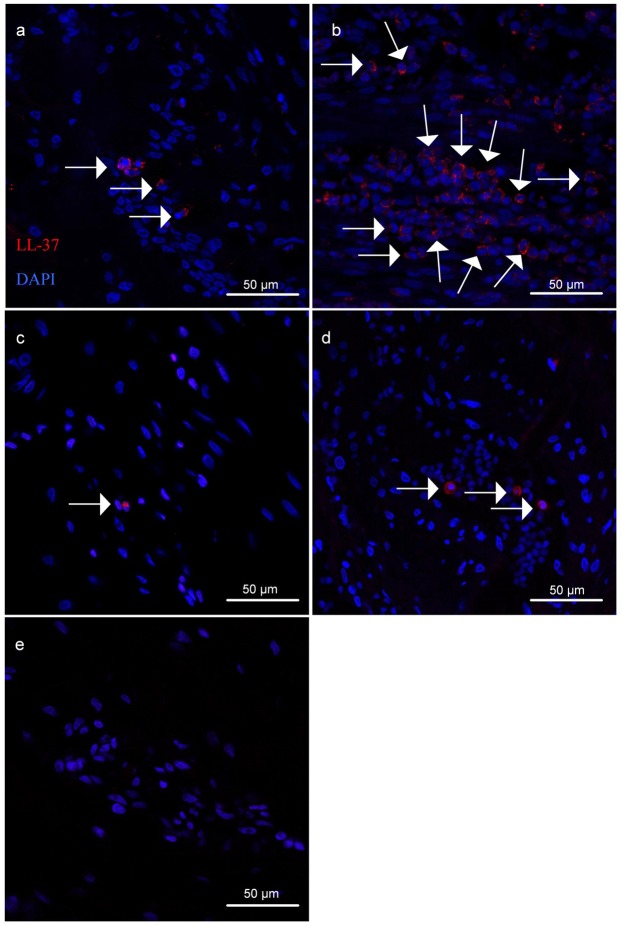
Representative images of LL-37 immunofluorescent staining used to determine percentage of LL-37 positive cells in TNL, TL, PTNL, and PTL myometrium. LL-37 positive cells (red) indicated by arrows in (**a**) TNL, (**b**) TL (**c**) PTNL, (**d**) PTL myometrium (**e**) Secondary antibody-only negative control.

The myometrial samples were analysed for *CAMP* gene expression as well as the percentage of LL-37 positive cells (by immunofluorescence) in relation to total number of nuclei counted. Although no differences were found in *CAMP* gene expression between the groups (Fig. 6a), the percentage of LL-37 positive cells present in the human myometrium was higher in TL samples in comparison to TNL (35.5% vs 2.02% p < 0.0001), PTNL (35.5% vs 2.44% p < 0.0001) and PTL (35.5% vs 2.66% p < 0.0001) myometrial samples (Fig. 6b). Representative images of LL-37 immunofluorescent staining used to determine the percentage of LL-37 positive cells are shown in Fig. 7.

## Discussion

In this study, we demonstrate that mouse cathelicidin, mCRAMP, may play a role in the onset of inflammation associated PTB stimulated by LPS. We show that mCRAMP and IL-6 are upregulated in the uterus 6 hours following a labour-inducing intrauterine LPS injection stimulus. *Camp*^−/−^ mice, which lack the mCRAMP protein, are less likely to go into labour following intrauterine LPS and show reduced levels of LPS-induced IL-6. We also show that human cathelicidin, LL-37, is highly expressed in myometrium from women who have had spontaneous labour at term, but we do not show this increase in myometrium from women who experienced preterm labour.

Cathelicidin is abundantly found in the reproductive tract^10^ and has been characterised as having both anti-inflammatory and pro-inflammatory roles^3^. Therefore, the role of this pleiotropic anti-microbial peptide in the context of PTB (well characterized as an inflammatory event)^16,24^, could be beneficial or detrimental. The direct microbicidal and anti-endotoxic properties of cathelicidins might be expected to be protective against PTB and the capacity of cathelicidin to bind to LPS and block activation of TLR4 might have been predicted to inhibit induction of PTB^3,25,26^. However, the level and timing of LPS and cathelicidin exposure is crucial to the response; cathelicidin is also capable of inducing inflammasome activation and IL-1β and IL-18 production in LPS-primed myeloid cells and suboptimally-stimulated, infected epithelial cells thereby contributing to an enhanced inflammatory response^27,28^. Furthermore, cathelicidin can act as a neutrophil chemoattractant^29^, thereby further enhancing inflammation^13^; highlighting the complexity and context-specificity of cathelicidin-mediated effects upon inflammation. Interestingly, it has been shown that endogenous mouse cathelicidin does not protect against LPS-induced shock, with similar or improved survival rates of *Camp*^−/−^ compared to wild type mice^30^, suggesting that anti-endotoxic properties of the endogenous peptide may not be the dominant effect in an *in vivo* inflammatory setting^31^.

**Figure 8:**
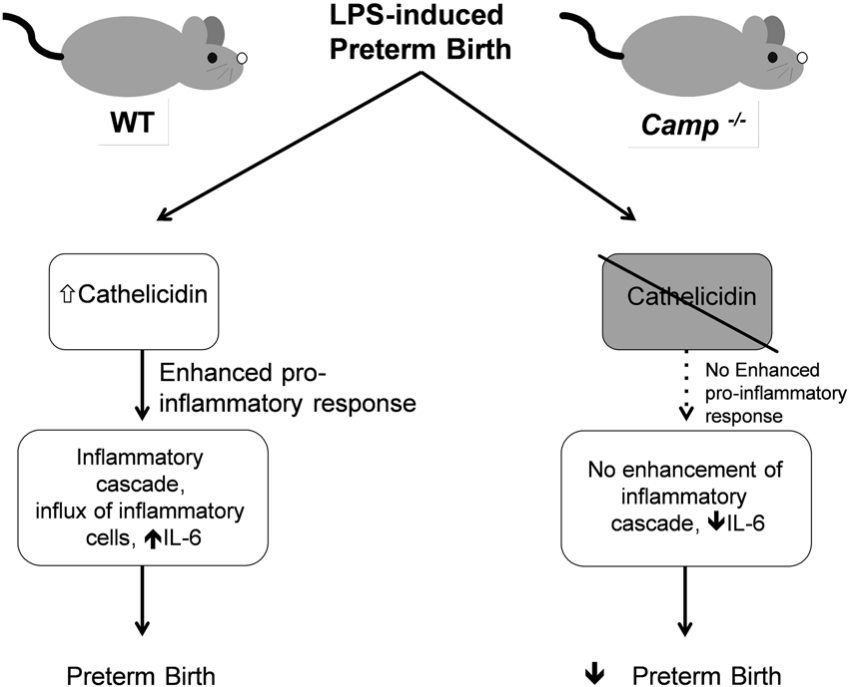
The role of cathelicidin in the onset of preterm labour triggered by infection and inflammation as modelled by an intrauterine LPS injection. mCRAMP is increased in the wild type mouse uterus following exposure to LPS. This increase mediates a pro-inflammatory response that leads to an enhanced inflammatory cascade, influx of inflammatory cells and an increase in IL-6 (left hand panel). This in turn leads to preterm parturition. In *Camp*^−/−^ mice, in the absence of mCRAMP, LPS fails to trigger the inflammatory cascade and an increase in IL-6 production. Consequently, *Camp*^−/−^ mice have a decreased rate of preterm labour in comparison to wild type mice following a low dose intrauterine LPS injection (right hand panel).

The pro-inflammatory effect of cathelicidin can be beneficial in certain circumstances. Indeed, we have shown that cathelicidin can promote pulmonary clearance of *Pseudomonas aeruginosa* in an *in vivo* murine model by enhancing the neutrophil response^13^. In our murine LPS-induced PTB model, intrauterine LPS injected *Camp*^−/−^ mice, compared to wild type mice, showed significantly less circulating maternal IL-6; a pro-inflammatory cytokine shown to increase in myometrial cells after LPS stimulation^32^ and known to play an important role in the development of preterm and term labour^33–35^. This may suggest that mCRAMP influences the inflammatory profile to some extent and may have the potential to trigger the inflammatory cascade that ultimately leads to labour (Fig. 8). However, it may contribute to the onset of PTB through alternative pathways as CXCL10 and TNF, also known pro-inflammatory cytokines, were found to be similar in both wild type and *Camp*^−/−^ mice following an intrauterine LPS injection. Transcription of *PTGS2* (encoding COX-2 enzyme), *CXCL1* (encodes the chemokine CXCL1 which regulates the recruitment of neutrophils and basophils during inflammation^36,37^) were also investigated in the mouse myometrium, however, these were found to be unaltered following 1 μg LPS injection at 6 hours.

Similar delivery rates were seen in wild type and *Camp*^−/−^ mice when a high concentration of LPS (20 μg) was administered, suggesting that a sufficiently strong inflammatory stimulus can override the protective effect of mCRAMP deficiency.

It has been previously shown that cathelicidin is increased in a variety of tissues in various inflammatory conditions, and upon LPS exposure^3,38–40^. This is consistent with our observations in the mouse uterus with mCRAMP significantly increased 6 hours after LPS injection. Although mouse pregnancy is considerably different from human pregnancy, studies have indicated that the mouse model can potentially be useful for preclinical studies of human labour^19,22,41^. In our study we used human myometrium as an initial insight into the expression of LL-37 in context of labour as it forms the majority of the uterine wall and is central to labour as it is responsible for uterine contractions. We observed an increase in LL-37 protein levels during human term labour, compared to non-labour. These findings are in line with those of Lim *et al*. who found an increase in LL-37 protein levels in human myometrium from term women in labour compared to term women undergoing a pre-labour caesarean section^42^. They found LL-37 localised to myometrial muscle fibres in both labouring and pre-labouring samples, and in labouring samples LL-37 was also found in leukocytes^42^. LL-37 is known to be predominantly produced by neutrophils and epithelial cells^3^ and as there is an influx of neutrophils in term and pre-term labouring myometrium^17,43^, we hypothesise that the LL-37 increase is at least in part due to this infiltration^20,21^. However, we were unable to confirm whether neutrophils were a source of the LL-37 increase in our human myometrial samples, due to technical failure of dual immunofluorescent staining on paraffin embedded tissues. To overcome this limitation in future experiments, collected samples could be frozen for staining on frozen tissue, which may work better in this type of analyses. Furthermore, flow cytometry could be utilised to identify the LL-37 positive infiltrate cells. Importantly, future investigations should also include the study of other reproductive tissues, such as the endometrium and the amnion/chorion, as inflammatory mechanisms vary in different tissues.

Despite a greater proportion of LL-37 positive cells present in labouring human myometrium, we did not observe an equivalent increase in myometrial *CAMP* gene expression, suggesting that the LL-37 we observe in these human samples may primarily be stored and released from granules, as opposed to being synthesized *de novo*. Indeed, LL-37 in neutrophils is known to be predominantly pre-synthesised and stored as pre-peptides in granules^9,44^. This may potentially explain the discrepancy between the levels of mRNA and of protein seen in the human labouring myometrium. This contrasts our findings in the mouse, where LPS stimulated the production of mCRAMP at both mRNA and protein level. We did not detect a difference in LL-37 levels in the human myometrium when we compared samples from women in preterm labour and those having pre-labour preterm delivery. As all preterm deliveries are abnormal, finding an appropriate control group for the study of gene and protein expression changes during preterm labour is notoriously difficult. Many of the conditions that indicated iatrogenic PTB in our control, pre-labour caesarean section preterm group, are themselves associated with inflammation (e.g. pre-eclampsia, placental insufficiency and abruption). Thus, the lack of a detectable difference may reflect increased inflammation in both groups. In the present study, we cannot exclude that the regulation of LL-37 in the myometrium is different in preterm labour compared to preterm pre-labour, future research is needed for this, using appropriate control groups which are difficult to obtain. However, there was less LL-37 protein expression in preterm labour samples compared to term labour samples which may indicate differences in the pathophysiology of preterm and term labour, or may reflect other differences between the groups (e.g. duration of labour at time of sampling). The lack of an LL-37 upregulation in myometrium of the PTNL women might be considered inconsistent with our findings in the mouse model where we see an upregulation of mCRAMP 6 hours post-LPS injection. However, a previous study has shown that changes in the transcriptome in mouse myometrium induced by intrauterine LPS injection mirror those in the human myometrium seen during term parturition more closely than those seen during preterm labour^19^. Our data supports this finding as we also see a parallel between human TL myometrium and mouse uteri exposed to LPS, in that both show an upregulation of cathelicidin. In the mouse term labour is initiated through progesterone withdrawal and is associated with much less inflammation^45^. This corresponds with our observation in preliminary experiments examining mCRAMP expression throughout mouse gestation, where we found no evidence of upregulation in term labour.

In conclusion, we show that LPS increases the expression of cathelicidin in wild type mouse uterus and an absence of cathelicidin in *Camp*^−/−^ mice reduces the incidence of PTB triggered by a low dose of LPS. In humans, cathelicidin protein levels are increased in myometrium undergoing term labour. These data suggest that cathelicidin plays a role in mediating some of the effects which lead to inflammatory PTB in mice, and may also be relevant in the onset of human labour.

## Methods

### *In vivo* experiments

*In vivo* studies were conducted under a UK Home Office license to J.E.N. (60/4241) in accordance with the Animals Scientific Procedures Act (1986). C57Bl/6 virgin female mice were purchased from Charles River Laboratories (Margate, UK). *Camp*^*tm1Rig*^ mice^46^ were backcrossed to congenicity onto a C57Bl/6J OlaHsd strain, and bred from homozygous matings in house in specific pathogen free facilities, in individually ventilated cages at the University of Edinburgh. Following matings, mice were housed in grouped cages of up to 6 mice per cage under specific pathogen free conditions at the same facility. Mice were permitted food and water as required and maintained in a 12-hour cycle of light and dark. All mice were acclimatized for a minimum of 1 week before use. Female mice were mated with stud males from the same genotype. The presence of a vaginal copulatory plug indicated day 1 of gestation. Pregnant mice with no appearance of a plug were not used in experiments, as gestational age was uncertain.

### *In vivo* preterm birth model

The ultrasound guided intrauterine injection mouse model of PTB previously optimised in our group was used^22^. A dose response curve using wild type mice was performed to determine the minimal dosage needed to induce PTB (1 μg/dam), which was used parallel with the highest dose previously used (20 μg/dam) to determine the effects on PTB rates of the different dosages, (Fig. S1). Differences in the inflammatory response were also investigated (Fig. S2). Space between two intra-amniotic sacs was located with ultrasound where the injection was administered. 25 μl of sterile DPBS or LPS (20 μg/dam or 1 μg/dam) was injected at gestational age day 17. After recovery, mice were monitored using closed circuit television cameras and a video recorder. Time of delivery was the time between injection and delivery of first pup. PTB was defined as delivery within 24 hours of intrauterine injection.

### *In vivo* timed collection experiments

A separate cohort of mice was used to assess the inflammatory effect of the stimuli used on maternal tissues. Mice were sacrificed by lethal exposure to carbon dioxide 6 hours after the intrauterine injection. A group of “no treatment” mice were also included. These mice were 17 days of gestation and received no treatment, and were culled in the same manner described above. Maternal serum was acquired by collecting whole blood through puncture of the aorta using 21G needles in Brand Tubes Microtainers tubes and spun for 5 minutes at 5.9 × *g* for ELISA analysis. Uterine samples were dissected and snap frozen in RNA-free tubes for protein and RNA analysis and separate samples were fixed in 4% neutral buffered formalin and embedded in paraffin blocks for immunofluorescence analysis.

### Enzyme-linked Immunosorbent Assay (ELISA)

EBiosceince duosets for IL-6 and TNF-α and an R&D Duoset for CXCL-10 was used for cytokine analysis. Duosets were used according to the manufacturer’s protocols.

### Protein extraction

Uterine samples were homogenised in RIPA Buffer (R0278, Sigma Life Sciences, Sigma-Alrich, UK) with a protease inhibitor (Protease Inhibitor Cocktail Tablets, Roche Diagnostics, IN, US) in a tissue lyser (Qiagen, Crawley, UK). Lysates were spun down at 4 °C for 10 minutes at 10,000 g. Protein was quantified using a Bio-Rad DC Protein Assay Kit (Bio-Rad Laboratories Ltd, Hertfordshire, UK).

### Western blot

The protein from the uteri samples was run at 40 µg on NuPAGE Novex 4–12% and Bis-Tris Gels (Invitrogen, Life Technologies Ltd, Paisley, UK). Protein was added to 2.5 µl of LDS Sample Buffer and 1 µl of Reducing Agent (Invitrogen, Life Technologies Ltd, Paisley, UK). Samples were denatured at 70 °C for 10 minutes on a G-Storm Thermocycler. A positive control of 0.1 µg of mCRAMP peptide was used. A Wet Blotting System (Bio-Rad Laboratories Ltd, Hertfordshire, UK) was used for the wet transfer of the protein in the gel to the Immobilon Fl PVDF membrane (Millipore, UK). The membrane was blocked for 1 hr in 5% Milk (Marvel, UK) in TBST (0.1%Tween in 1 L TBS). Rabbit-Anti-CRAMP antibody (Innovagen, Lund, Sweden) was used at a 1:100 dilution. Mouse-Anti-α-Tubulin (Sigma, T9026) at a 1:5000 dilution was included as a loading control. Fluorescent secondary antibodies (Polyclonal Donkey Anti-Rabbit 800CW (926-32213) and Polyclonal Donkey Anti-Mouse 680RD (926-68072), LICOR Biosciences) were used in 10 ml of 5% Milk in TBST. Signal detection was through the use of LI-COR Odyssey Infrared Imaging System. The intensity of fluorescence in each lane was calculated using Image Studio Version 4.

### Collection of human myometrial samples

Myometrium was collected from the inferior margin of the incision site of women delivering by caesarean section, at the Royal Infirmary, Edinburgh. Written and informed consent was obtained according to the ethical approval and governance granted to the Edinburgh Reproductive Tissues BioBank by the West of Scotland Research Ethics Committee 4 (REC reference: 09/S0704/3) until 30/09/2014; and consequently by the East of Scotland Research Ethics Service Tayside Committee on Medical Research Ethics B (REC reference:13/ES/0126). Upon collection, samples were placed into RNAlater solution (R0901 Sigma-Aldrich) for 24 hours at 4 °C, then taken out and stored at −80 °C prior to use. Inclusion criteria was singleton pregnancies that were term (>37 weeks of gestation) or preterm (<37 weeks of gestation) whilst exclusion criteria were age under 16 and any blood borne infections. Women either underwent an elective pre-labour caesarean section (no labour group) or an emergency CS in labour due to maternal and/or fetal indications (e.g. delay in labour, pre-eclampsia, fetal distress). Labour was defined as regular uterine contractions with cervical dilation.

### qRT-PCR

Total RNA was extracted using the Rneasy Mini Kit (Qiagen, Crawley, UK) as per the manufacturer’s guidelines and converted into cDNA using the High Capacity cDNA Reverse Transcription Kit (Applied Biosystems, Life Technologies Ltd, Paisley, UK) in a G-Storm GS1 Thermocycler (G-storm, Somerton, UK). Taqman Gene Expression Assay was used to determine gene expression, with a TaqMan Universal Mastermix (Applied Biosystems, Life Technologies, Paisley, UK). *CAMP (Hs00189038*_*m1)*, *ACTB (431088E)*, *Camp (Mm00438285*_*m1)*, *Ptgs-2 (Mm00478374*_*m1)*, *Cxcl1 (Mm04207460*_*m1)*, *Tnf (Mm00443258*_*m1)*, *Actb (4352341E)* probes Applied Biosystems, Life Technologies, Paisley, UK) were used. The qRT-PCR was performed on the ABI 7900Ht (Applied Biosystems, Carlsbad, CA). Gene expression was determined using the ΔΔCt method, relative to housekeeping gene *ACTB*.

### Immunofluorescence

Paraffin-embedded uterine sections on slides were dewaxed in xylene and rehydrated in decreasing alcohols to 70%. For LL-37 and mCRAMP detection, antigen retrieval was undertaken (0.1 M Citrate Buffer; decloaking chamber). Peroxidase blocking was carried out for 30 minutes (300 mls of 0.1% H_2_O_2_, in methanol) followed by a 30 minute serum block (Normal Goat Serum (NGS) in TBS with 5% BSA). Primary antibodies 1:10 LL-37 (Cayman; CAY 15637), 1:2500 mCRAMP (Abcam -ab74868) and 1:4000 Ly6G (Biolegend; 127602) was incubated on slides in a hydrated slide tray overnight at 4 °C. Negative controls were performed using NGS in place of the primary antibody. After two TBS washes, secondary antibodies were incubated on the slides for 30 minutes with goat anti-Rabbit AF555 (A-21429, Thermo Fisher Scientific, 10 µg/ml) Immpress Anti-Rabbit and Immpress Anti-Rat (Vector Laboratories Ltd, Peterborough, UK) for LL-37, mCRAMP and Ly6G respectively. Tyramide Signal Amplification (TSA; Tyramide Blue (TSA™-Plus Cyanine 5 System, NEL745B00KT, Perkin Elmer, MA, US), 1:50 for mCRAMP and Tyramide Red (TSA™-Plus Cyanine 3 System, NEL744B00KT, Perkin Elmer, MA, US), 1:50 for Ly6G and counterstaining for nuclei (Sytox Green (S7020, Invitrogen Molecular Probes, Life Technologies Ltd, Paisley, UK), 1:1000). Mouse uteri slides were mounted with permafluor (Thermo Scientific, UK) and human myometrium slides were mounted with Vectashield containing DAPI (Vector Laboratories Ltd, Peterborough, UK) with for confocal analysis.

### Counting % of LL-37 positive cells

Images at Magnification of x40 were captured on the LSM710 confocal microscope. At least five areas were imaged from each sample. Four individual patient samples were analysed from the TL group and 5 patient samples were analysed from the TNL group. Image files were then imported into ImageJ and analysed using the Cell Counter plug-in. LL-37 positive cells were counted in each section and expressed as a percentage of total cells in section which was counted as total number of DAPI nuclei in the section.

### Statistical analysis

Statistical analysis was carried out on GraphPad Prism version 7 (GraphPad Software, San Diego, California, US), with data presented as mean ± SD. Time to delivery data, mouse/human gene expression, cell count and ELISA data were analysed using a two-way ANOVA followed by Tukey’s post-hoc analysis. PTB rate was calculated using Fisher’s exact test, where PTB was defined as delivery less than 24 hours after intrauterine injection. Mouse data in which mCRAMP expression was quantified, was analysed using a one-way ANOVA. p < 0.05 was considered as statistically significant in all analyses.

## Supplementary information

Supplementary Information

## Data Availability

The datasets generated during and/or analysed during the current study are available from the corresponding author on reasonable request.
