# Supplementary material for: Cathelicidins and the Onset of Labour
Source: Sci Rep. 2019 May 14;9:7356. doi: 10.1038/s41598-019-43766-7 (PMC6517412; doi:10.1038/s41598-019-43766-7)
Supplement: Supplementary file 1 — Supplementary Information [file 41598_2019_43766_MOESM1_ESM.pdf]

Cathelicidins and the Onset of Labour

Sara R van Boeckel[1†], Lenka Hrabalkova[1†], Tina L Baker[2†], Heather MacPherson[1], Lorraine Frew[1], Ashley K Boyle[1], Brian J McHugh[2], Kirsten Wilson[1], Jane E Norman[1], Julia R Dorin[2], ***Donald J Davidson[2§], Sarah J Stock[1*§]***

[1] Tommy's Centre for Maternal and Fetal Health at the MRC Centre for Reproductive Health, University of Edinburgh, QMRI, Edinburgh, United Kingdom. [2] Medical Research Council Centre for Inflammation Research at the University of Edinburgh, QMRI, Edinburgh, United Kingdom.

Correspondence and requests for materials should be addressed to S.J.S. (Sarah.Stock@ed.ac.uk).

[†] *Indicates shared co-first authorship*

[§] *Indicates equal contributions and shared co-senior authorship*

*Corresponding author: Dr Sarah J Stock, Sarah.Stock@ed.ac.uk

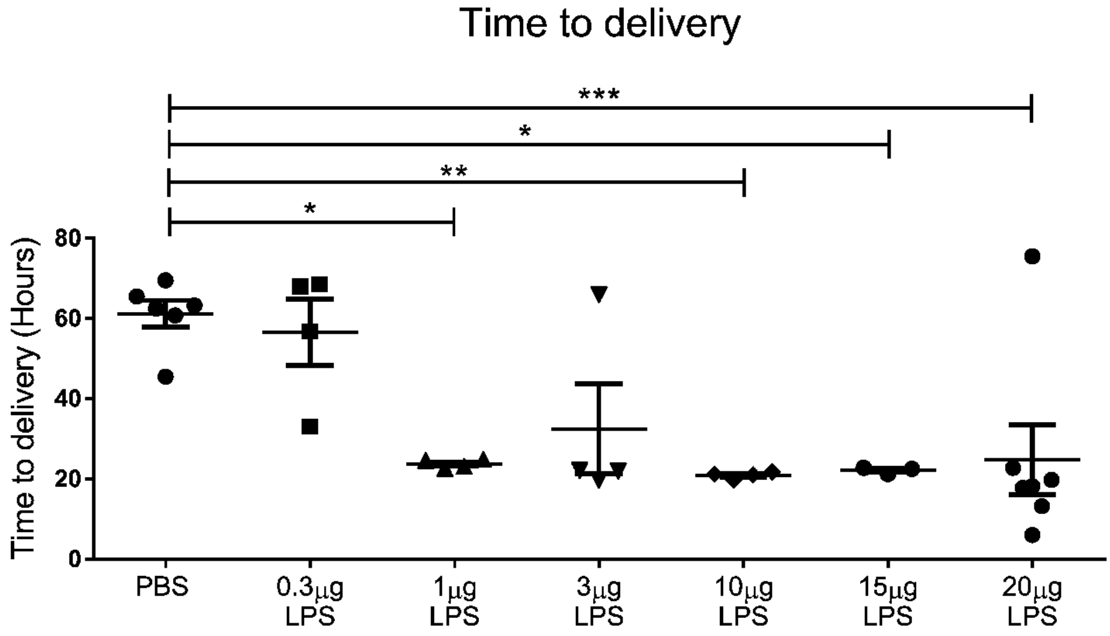

Supplementary Figure S1. Time to delivery. Mice received intrauterine PBS or LPS (0.3-20 μg) to induce preterm birth. An intrauterine injection of 0.3 μg LPS did not result in preterm labour, however, an injection of 1, 10, 15 and 20 μg of LPS significantly reduced the time from intrauterine injection to the delivery of the first pup compared to the PBS group. n=3-7, *p>0.05, **p>0.01. ***p>0.001, mean ±SEM, one-way ANOVA with Dunnett's post hoc test.

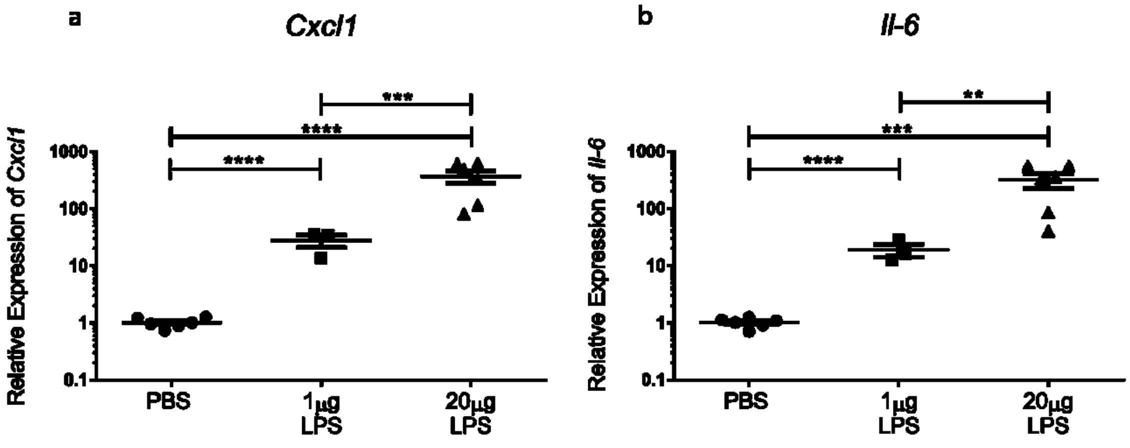

Supplementary Figure S2. Inflammatory gene expression in the uterus following and intrauterine injection of 1 μg and 20 μg LPS. (A) *Cxcl1* gene expression is significantly upregulated following the injection of LPS compared to a PBS injection, with 20 μg eliciting a greater response than 1 μg. (B) Similarly, the gene expression of Il-6 is also increased in response to LPS with 20 μg also eliciting a greater response than 1 μg. n=3-6, ***p>0.001, ****p>0.0001, mean ±SEM, one-way ANOVA with Tukey's post hoc test.

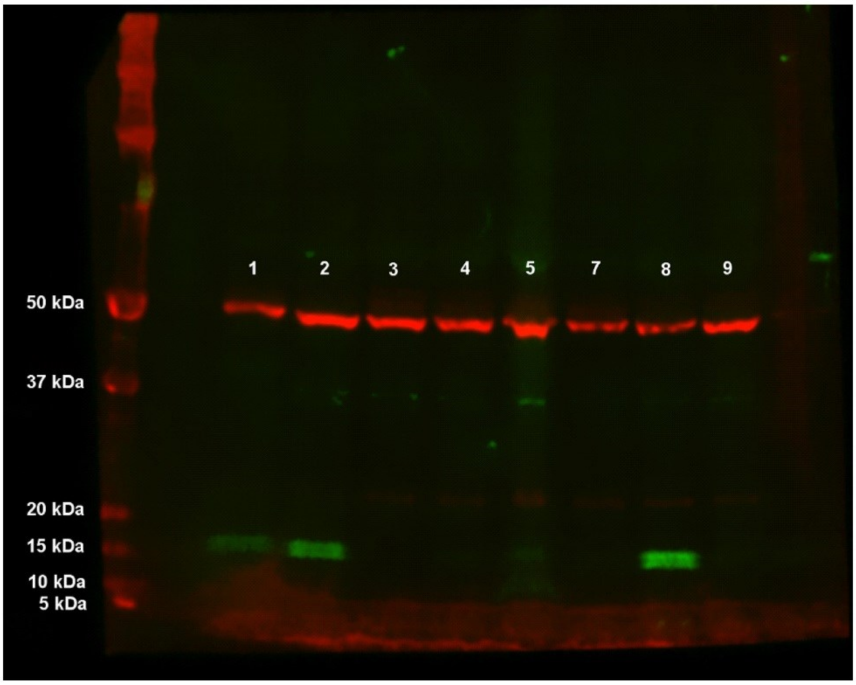

Supplementary Figure S3. Western blot showing changes in uterine mCRAMP expression following 1 µg LPS injection. Alpha-Tubulin was used as a loading control. Lanes 1, 2, 8 = LPS, lanes 3-7, 9 = PBS.

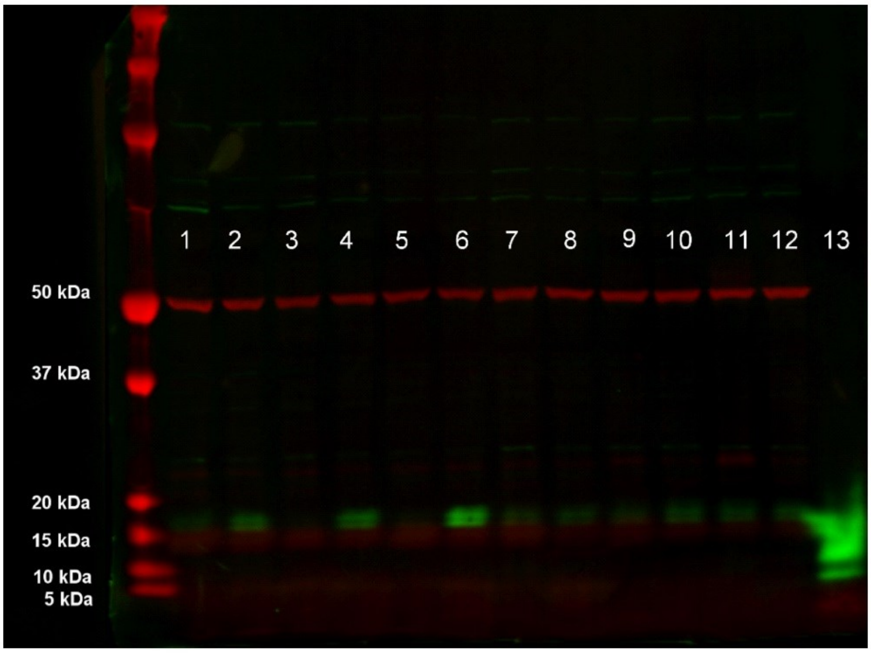

Supplementary Figure S4. Western blot showing changes in uterine mCRAMP expression following 20 µg LPS injection. Alpha-Tubulin was used as a loading control. Lanes 2, 4, 6, 7, 8, 10, 12 = LPS, lanes 1, 3, 5, 7, 9, 11 = PBS. Lane 13 is positive control of 0.1 µg of cleaved mCRAMP peptide (~5kDa).
